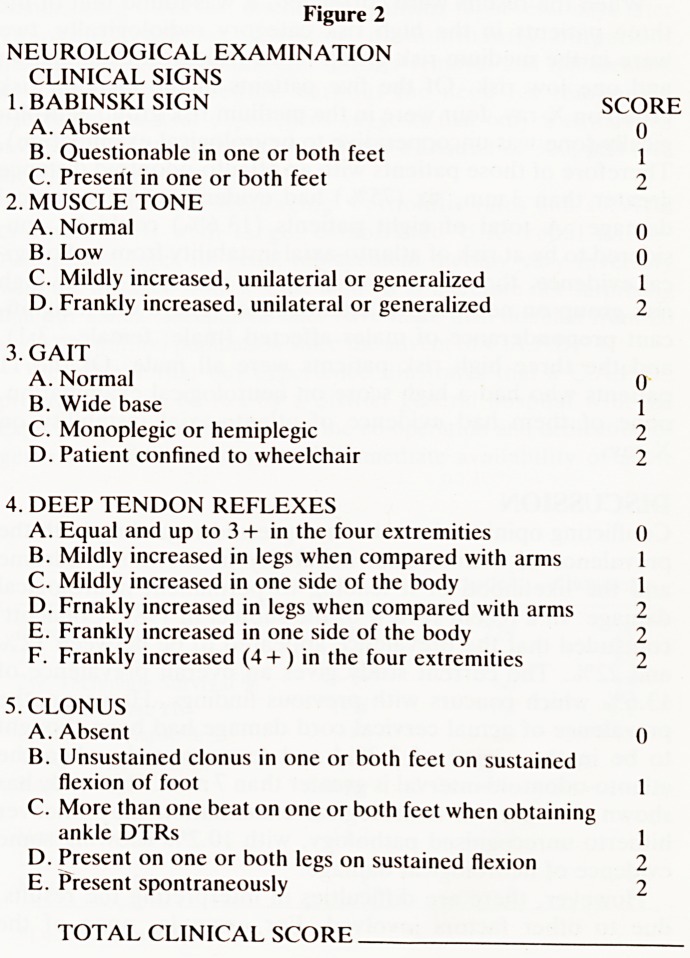# Atlanto-Axial Instability in Adults with Down's Syndrome

**Published:** 1991-03

**Authors:** Leila B. Cooke, R. Lansdall-Welfare

**Affiliations:** Consultant Psychiatrist, Stoke Park Hospital, Stapleton, Bristol; Consultant Psychiatrist, Highbury Hospital, Bulwell, Nottingham

## Abstract

A survey was performed on the patients with Down's syndrome living in three long-stay hospitals for the mentally handicapped in Bristol. Each patient had a chromosomal analysis, lateral X-rays of the cervical spine in flexion and extension and subsequent measurement of the atlantoodontoid distance, and a neurological examination. Out of a total of 59 patients, with ages ranging from 23 to 65 years (average age 48.27 years), three (5%) were found to be in the high risk category radiologically, but of these two were in the medium risk group on neurological examination and one low risk. Of five (8.5%) patients in the medium risk group on X-ray, four were in the medium risk group neurologically.

Therefore a total of eight patients (13.6%) could be considered to be at risk of atlanto-axial instability.


West of England Medical Journal Volume 106 (i) March 1991

### Atlanto-axial Instability in Adults with Down's Syndrome—A Survey of a Long-stay Hospital Population

*Leila B. Cooke, MB, ChB, MRC Psych
Consultant Psychiatrist, Stoke Park Hospital, Stapleton, Bristol

R. Lansdall-Welfare, MB, ChB, MRC Psych
Consultant Psychiatrist, Highbury Hospital, Bulwell, Nottingham

### SUMMARY

A survey was performed on the patients with Down's syndrome living in three long-stay hospitals for the mentally handicapped in Bristol. Each patient had a chromosomal analysis, lateral X-rays of the cervical spine in flexion and extension and subsequent measurement of the atlanto-odontoid distance, and a neurological examination. Out of a total of 59 patients, with ages ranging from 23 to 65 years (average age 48.27 years), three (5%) were found to be in the high risk category radiologically, but of these two were in the medium risk group on neurological examination and one low risk. Of five (8.5%) patients in the medium risk group on X-ray, four were in the medium risk group neurologically.

Therefore a total of eight patients (13.6%) could be considered to be at risk of atlanto-axial instability.

**Figure 1**

Laterial view of atlanto-axial subluxation in patient with Down's syndrome

### INTRODUCTION

The association of atlanto-axial instability with Down's syndrome has been recognised since 1961[1] but has received a revival of interest in the last few years. This may be because a policy of community care provides greater opportunites for participation in sporting activities in which the condition could prove dangerous.

Following the death of one of our patients from quadraplegia caused by atlanto-axial subluxation (Fig. 1), and because of the conflicting evidence on the prevalence and significance of this condition, we decided to undertake a large-scale survey of a hospital inpatient population.

### METHOD

All the patients in three long-stay hospitals for the mentally handicapped who had been diagnosed as having Down's syndrome were identified. Each patient had a chromosomal analysis performed, and lateral X-rays of the cervical spine in flexion and extension were taken. These were interpreted by consultant radiologist and the atlanto-odontoid distance measured. The patients also underwent a neurological examination performed by one of the authors (RLW). This was scored using the five parameters developed by Alvarez and Rubin[2], and was performed without knowledge of the radiological findings.

Each patient was then categorised as high, medium or low risk according to the radiological and neurological findings.

**Figure 2**

| NEUROLOGICAL EXAMINATION CLINICAL SIGNS | SCORE |
|---|---|
| 1. BABINSKI SIGN | |
| A. Absent | 0 |
| B. Questionable in one or both feet | 1 |
| C. Present in one or both feet | 2 |
| 2. MUSCLE TONE | |
| A. Normal | 0 |
| B. Low | 0 |
| C. Mildly increased, unilateral or generalized | 1 |
| D. Frankly increased, unilateral or generalized | 2 |
| 3. GAIT | |
| A. Normal | 0 |
| B. Wide base | 1 |
| C. Monoplegic or hemiplegic | 2 |
| D. Patient confined to wheelchair | 2 |
| 4. DEEP TENDON REFLEXES | |
| A. Equal and up to 3+ in the four extremities | 0 |
| B. Mildly increased in legs when compared with arms | 1 |
| C. Mildly increased in one side of the body | 1 |
| D. Frnakly increased in legs when compared with arms | 2 |
| E. Frankly increased in one side of the body | 2 |
| F. Frankly increased (4+) in the four extremities | 2 |
| 5. CLONUS | |
| A. Absent | 0 |
| B. Unsustained clonus in one or both feet on sustained flexion of foot | 1 |
| C. More than one beat on one or both feet when obtaining ankle DTRs | 1 |
| D. Present on one or both legs on sustained flexion | 2 |
| E. Present spontraneously | 2 |
| TOTAL CLINICAL SCORE ______ | |

### RESULTS

From a total of 740 patients in three hospitals, 59 (8%) were diagnosed as having Down's syndrome. Their ages ranged from 23 to 65 years (average age 48.3 years) and they were all moderately or severely mentally handicapped. The sexes were evenly divided.

Chromosomal analysis was performed on 48 patients; the remainder were either uncooperative to venepuncture or died or were discharged before it could be performed. Out of the 48, 44 had trisomy 21, one a translocation and three were mosaics.

Radiological examination was possible in 48 patients. Of these three were found to be in the high risk group (atlanto-odontoid distance >5 mm). Five were in the medium risk group (atlanto-odontoid distance 3–5 mm) and 39 were low risk (atlanto-odontoid distance <3 mm). (Table 1).

**Table 1**

**Results of radiological examination**

| | *Low risk (<3 mm)* | *Medium risk (3·5 mm)* | *High risk (>5 mm)* | *Total* |
|---|---|---|---|---|
| Males | 18 | 3 | 3 | 24 |
| Females | 22 | 2 | 0 | 24 |
| Total | 40 | 5 | 3 | 48 |

Neurological examination was performed on 49 patients, and each was given a score according to the five parameters developed by Alvarez and Rubin (Fig. 2). 11 patients scored two or more, which put them into the high risk category, 21 were medium risk, and 17 low risk. (Table 2).

**Table 2**

**Results of neurological examination**

| | *Low risk (0)* | *Medium risk (1)* | *High risk (2+)* | *Total* |
|---|---|---|---|---|
| Males | 9 | 9 | 5 | 23 |
| Females | 8 | 12 | 6 | 26 |
| Total | 17 | 21 | 11 | 49 |

When the results were correlated, it was found that of the three patients in the high risk category radiologically, two were in the medium risk group on neurological examination and one low risk. Of the five patients in the medium risk group on X-ray, four were in the medium risk group neurologically (one was uncooperative to neurological examination). Therefore of those patients with an atlanto-odontoid distance greater than 3 mm, six (75%) had evidence of neurological damage. A total of eight patients (13.6%) could be considered to be at risk of atlanto-axial instability from radiological evidence, though none of them are currently in the high risk group on neurological examination. There was a significant preponderance of males affected (male: female—3:1), and the three high risk patients were all male. Of the 11 patients who had a high score on neurological examination, none of them had evidence of atlanto-axial instability on X-ray.

### DISCUSSION

Conflicting opinions have been expressed regarding both the prevalence of atlanto-axial instability in Down's syndrome and the likelihood of it leading to permanent neurological damage. In a recent review of the subject in 1987, Collacott[3] concluded that the prevalence appeared to be between 12% and 22%. The current study gives an overall prevalence of 13.6% which concurs with previous findings. However, the prevalence of actual cervical cord damage had been thought to be in the region of 2.3%[4] and to occur only when the atlanto-odontoid interval is greater than 7 mm. This study has shown that careful neurological examination may uncover hitherto unrecognised pathology, with 10.2% showing some evidence of neurological damage.

However, there are difficulties in interpreting the results, due to other factors involved. For example, none of the patients in the high risk category neurologically had radiological evidence of atlanto-axial instability so their neurological damage must have had other causes. Six of them had evidence of cervical spondylosis, and one had had a previous cardio-vascular accident. This demonstrates the necessity for radiological screening, as one cannot rely on clinical examination alone in patients who may have multiple pathology.

Although the numbers involved are small, the preponderance of males affected is significant, and has not been noted in previous studies. Indeed, the opoposite has been the case[4]. The reasons for this are not known.

An interesting finding not directly relevant to the study is the high prevalence of cervical spondylosis and other degenerative changes uncovered. This occurred in patients in the 40–65 age range, with one patient aged 37 involved, thus providing more evidence of the premature ageing process in people with Down's syndrome.

People with Down's syndrome, in common with all those with a mental handicap, now have many more opportunities for leading as 'normal' a life as possible. This may mean they are participating in activities hitherto not open to them e.g. some sports, that they travel a lot more than previously, and that they may be more likely to require anaesthesia for operations. All these activities may lead to cervical cord damage in a person with undiagnosed atlanto-axial instability. Although some degree of risk taking may be unavoidable, even necessary, it seems that the problem of atlanto-axial instability is one area in which we can minimise the risk by screening. The findings in this study support the necessity for this.

### ACKNOWLEDGEMENTS

We would like to thank Mr R. Black, Senior Radiographer for taking the X-rays and Dr J. Fowles, Consultant Radiologist for interpreting them, and Dr J. Jancar for his advice in the preparation of the paper.

### REFERENCES

1. SPITZER, R., RABINOWITCH, J. Y., WYBAR, K. C. (1961). A study of the abnormalities of the skull, teeth and lenses in mongolism. *Canadian Medical Association Journal,* **84**, 567–72.
2. ALVAREZ, N., RUBIN, L. (1986) Atlanto-axial instability in adults with Down's syndrome: a clinical and radiological survey. *Applied Research in Mental Retardation,* **7**, 67–78.
3. COLLACOTT, R. A. (1987) Atlanto-axial instability in Down's syndrome. *British Medical Journal,* **294**, 988–89.
4. PEUSCHEL, S. M., HERNDON, J. H., GELCH, M. M., SENFT, K. E., SCOLA, F. H., GOLDBERG, M. J. (1984). Symptomatic atlanto-axial subluxation in persons with Down's syndrome. *Journal of Paediatric Orthopaedics,* **4**, 682–88.

* Correspondence: Dr. Leila B. Cooke, Stoke Park Hospital, Stapleton, Bristol BS16 1QV.

## Figures and Tables

**Figure 1 f1:**
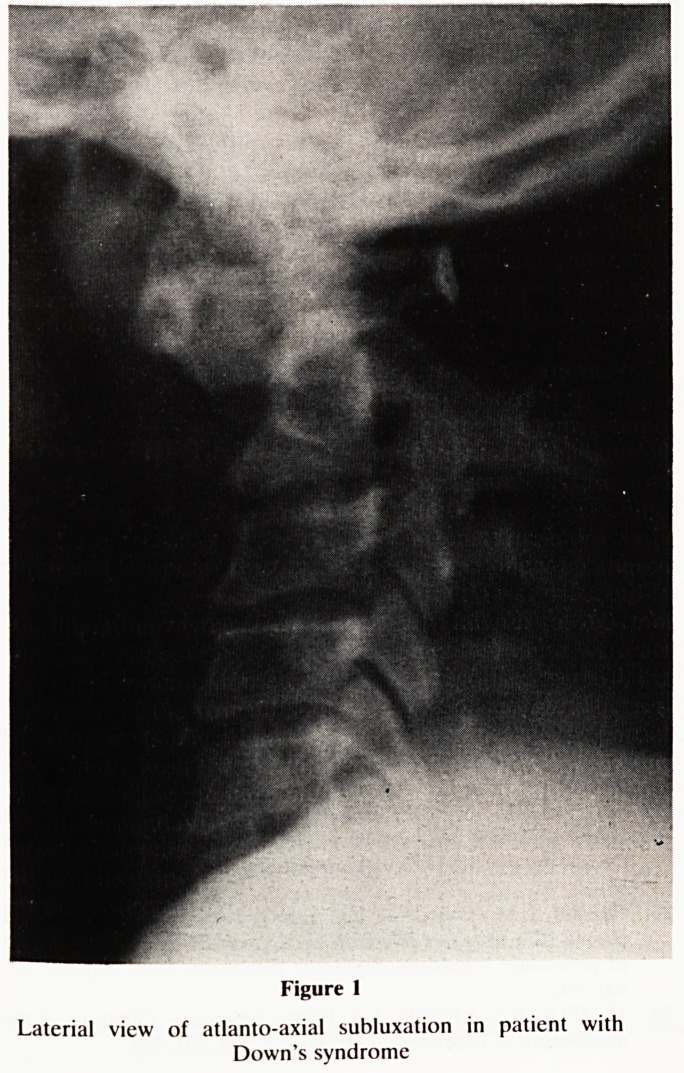


**Figure 2 f2:**